# Use of dental care services among adolescents living with HIV on Antiretroviral Treatment in Kampala, Uganda: a cross-sectional study

**DOI:** 10.21203/rs.3.rs-3833085/v1

**Published:** 2024-01-25

**Authors:** Maria Gorretti Nakyonyi, Nancy Birungi, Catherine Lutalo Mwesigwa, Anne Nordrehaug Åstrøm

**Affiliations:** Center of International Health, University of Bergen; Oral health Centre for expertise in Western Norway; School of Dentistry, Makerere University; Institute of Odontology, University of Bergen-department of Global Oral health

**Keywords:** Use of dental care services, HIV, ART, adolescents, Kampala, Uganda

## Abstract

**Objective:**

The main purpose of this study to assess the prevalence and socio-behavioural determinants of ever-use of dental care services among the adolescents aged 10–18 years, living with HIV on Antiretroviral treatment (ART), attending selected HIV clinics in Kampala, Uganda.

**Methods:**

A cross-sectional study was carried out between March and September 2020. The study conveniently recruited 154 adolescents between 10–18 years from 4 specific HIV clinics in Kampala. The Andersen’s behavioral model guided the selection of variables in terms of ever use of dental care services as the outcome- and predisposing, enabling, need related factors and dental health related behavior as exposure variables. Data was analyzed using Fischer’s exact test for cross-tabulation and modified Poisson regression for multivariate analysis.

**Results:**

The prevalence of ever-use of dental care services was 12.3%. The adolescents aged 14–18 years were more likely to have used dental care services (Prevalence ratio (PR) of 3.35 (Confidence Interval (CI) 1.48–7.59) than those aged 10–13 years. Fear of spread of HIV was negatively associated with ever-use of dental care services (PR of 0.06 and CI of (0.01–0.44). Participants who were afraid of going to the dentist were more likely to have ever used dental care services (PR of 2.98 and CI of 1.41–6.30) than those not afraid. Failure to receive dental treatment because it was not part of the medical appointment had a positive association with ever-use of dental care services (PR of 4.50 (CI: 1.14–17.80). Those who were satisfied with their dental condition were less likely to have ever-used dental care services (PR of 0.21 and CI of (0.05–0.94). Bad oral odor was positively associated with ever-use of dental care services with a PR of 2.80 and CI of 1.19–6.60. Use of soap for toothbrushing was positively associated with ever-use of dental care services (PR of 2.51, CI of 1.47–4.28).

**Conclusion:**

The study found low frequency of dental care use among HIV infected adolescents in Kampala, Uganda, with age being a predisposing factor. Enabling factors included fear of HIV spread, dental appointment failure, and satisfaction with dental condition and bad oral odor while under personal oral hygiene and dental practices, use of soap for toothbrushing was an important association of use of dental care.

## Background

The Joint United Nations Program of HIV and AIDS (UNAIDS) reports that two of every seven new HIV (Human Immunodeficiency Virus) infections are among the young people (15–24 years). (1). The United Nations International Children’s Emergency Fund (UNICEF) reported that of the 400,010 HIV estimated incidence cases, 150,000 of these were adolescents aged 10–19 years. (2). Adolescents and young people are still heavily affected by HIV, accounting for 37% of all new infections in 2017 as well as 15% of all people living with HIV (3). The Uganda Population-Based HIV Impact Survey (UPHIA) of 2022 indicated that the current prevalence of HIV is 5.5% among the Ugandan adult population and 1.8% among those aged between 15–24 years (4). According to the HIV Investment framework for Uganda 2021-30, approximately 65% children between 0 and 14, living with HIV are currently on Antiretroviral treatment (ART) and among those above 15 years, about 85% were on ART as of 2019 (5).

Oral health care is an integral part of HIV care and the spectrum of HIV-associated opportunistic diseases occurring in the oral cavity propelled dental health care providers to the forefront of patient care (6). Over 90% of individuals living with HIV will at least have one oral manifestation attributed to HIV infection during the course of life (7).

The most common oral manifestations include pseudomembranous candidiasis, angular cheilitis, necrotizing ulcerative gingivitis (NUG) and necrotizing ulcerative periodontitis (NUP) oral hairy leukoplakia, Kaposi sarcoma, human papilloma virus oral warts, common ulcerative conditions, and dental caries (8-10). Oral diseases may also cause symptoms such as pain, discomfort, altered taste, and a burning sensation (11). Additionally, oral symptoms may interfere with the daily functions of life such as chewing of food, pronunciation of certain words and sounds, and smiling and socializing with confidence (11).

Following the advent of ART, there has been a decrease in the prevalence of HIV-related oral lesions of 10–50% (12). However, Scully et al, reported undesirable effects of ART on oral health (13). Antiretroviral drugs have been reported to have side effects like xerostomia, oral lichen lesions, erythema multiform from drugs like Didanosine and Zidovudine (13). Indinavir has had reports of causing angular cheilitis (13). Xerostomia is known for increasing the risk of dental caries and hence people living with HIV remain burdened with oral health concerns (14).

Oral health problems can significantly compromise general health and well-being of individuals living with HIV, and yet many of them experience an unmet need for dental care (15). Dental care has been reported to be one of the greatest unmet health care needs among individuals living with HIV (16). Given that the incidence and severity of dental disease in individuals living with HIV is greater than in the general population, available and accessible oral health care is especially important in this population (6). A study done in Uganda, reported a caries prevalence of 80% in adolescents living with HIV taking ART compared to 67% in the general adult population (17). Another study carried out in Uganda reported that individuals living HIV and taking ART had a need of dental treatment of about 96% and a DMFT (Decayed, Missing, Filled Teeth) score of four (14). People living with HIV (PLHIV) are now living longer due to ART, which means that oral diseases, such as dental caries and other oral manifestations are increasingly important to manage in this population. Therefore, there is a need for HIV infected patients to have easy access to dental care.

Andersen’s behavioural model has been a commonly used health theoretical tool used in assessing use of health care services including dental care services across several studies [37–48]. Uganda has a limited number of dentists, with only 300 serving the 44 million population. Private ownership makes dental clinics expensive, especially for marginalized groups (18). The country lacks an HIV dental integrated healthcare system. A 2020 study revealed that 15% of Uganda's rural areas lack a public dental facility out of 97 districts (19). Previous studies of oral health in PLHIV conducted in Uganda have focused on the oral health related quality of life and oral manifestations in this category of patients (20-24). No known s study has identified covariates pertaining to ever use of dental health care services among young people (adolescents) living with HIV.

The main purpose of this study to assess the prevalence and socio-behavioural determinants of ever-use of dental care services among the adolescents living with HIV on ART, between 10–18 years of age, attending selected HIV clinics in Kampala, Uganda.

## Methods

### Study design, setting and participant recruitment

This was a cross-sectional study carried out between March and September 2020. It was a sub study of a larger study (hereby called parent study) entitled *“Oral health quality of life and dental treatment needs among HIV + children and adolescents on ART attending selected HIV clinics in Kampala.”* The study was carried out in Kampala, the capital city of Uganda. The participants were selected from four HIV clinics: Mulago Immune Suppressive Syndrome (ISS) clinic, Kawaala Health Centre IV, Kisenyi Health Centre IV, and Kiswa Health Centre III which acted as clusters. The sampling frame for this sub study was 400 adolescents aged 10–18 years as of March to September 2020 attending the four selected HIV clinics. Sample size was estimated using an online calculator at 95% confidence level, significance level 0.05, the calculation was based on the premise that estimated population proportion of HIV infected adolescents that have ever used dental care was 50% (25). The minimum sample size that was required for this study was 197 adolescents (25). A total of 246 adolescents were conveniently contacted according to availability of their telephone contacts and consent to participate in a telephone interview. Of these 44 participants had either wrong telephone numbers or their numbers were inaccessible. These 44 were excluded from the study and not included in the analysis. Another 48 participants were excluded from the study because they were not aware of their HIV serostatus. A total of 154 adolescents were included in the study and hence the participation rate was 62.6% (154/246).

Inclusion criteria for this sub-study included participants had to be part of the parent study and 10–18 years as of March to September 2020. They had to be HIV positive, aware of the HIV serostatus and attending HIV clinics Kawaala, Mulago, Kisenyi, and Kiswa, and whose telephone contacts were accessible and available at the time the study was conducted.

### Data collection, quality management and storage

A pilot study was conducted involving 15 participants aged 10 to 18 years (not included among the 154 adolescents that officially participated in the main study).

### Interviews

This study was strictly based on telephone interviews due to the COVID-19 rules and guidelines (26). Each interview lasted for about 30 minutes. A structured questionnaire that guided the interview was used using the Open Data Kit form (27) that had been standardized for all participants. Two research assistants underwent a face-to-face training in using the Open Data Kit form and they were in addition trained on how to conduct telephone interviews in both English and Luganda. They were each given a description of the study objectives and study outcome for standardization of the tool. They were both given electronic tablets Samsung Galaxy SM- T285 S# R52J60MZ22V and R52JB215X8TA and trained how to use them. Both these devices were loaded with the standardized questionnaire in an ODK form and was used uniformly for all study participants.

The questionnaire included socio-demographic characteristics, factors affecting use of dental care services, personal dental health practices, general and HIV related concerns and attitudes and included the outcome variable “ever-visit- the dentist”.

Parents or caregivers were asked if the child was aware of their HIV serostatus at the start of interviews to exclude those not aware, as some parents withheld information about the serostatus and explained other reasons for frequent treatment. The questionnaire was translated to Luganda from English by a local translator. Later five independent people (the main project study investigator, 2 research assistants and 2 pilot study participants) reviewed the accuracy of the language translation. Back translation from Luganda back to English was carried out informally through analysis of questions if they meant the same in English. Therefore, the telephone interviews were conducted mostly in the locally spoken language, Luganda, and a few in English. Andersen’s behavioural model guided the selection of variables used to determine access and use of dental services among the selected group of participants (28)

## Measurements

### Dependent variable

The dependent variable of this study was ever-use of dental services which was phrased as “ever-visit the dentist” in the questionnaire. The participants were asked if they had ever visited the dentist with exception of the project participation. Response categories were given as yes and no. Those who confirmed dental attendance were followed up with the question “when was your last visit to the dentist?” - response were given as (1) less than 6 months ago, (2) 6–12 months ago, (3) more than a year but less than 2 years ago, (4) 2–5 years ago, (5) more than 5 years ago.

### Independent variables

The various variables were defined according to Andersen’s behavioral model into predisposing (socio-economic) factors, enabling factors, need related factors, oral hygiene, and personal health practices.

Among predisposing factors, gender was categorized into boys (0) and girls (1); age was grouped into 2 groups; 10–13 years (1) or 14–18 years (2). The level of education had 6 options, and these included: Primary 1–3 (1), Primary 4–7 (2), Senior 1–4 (3), Senior 5 or 6 (4), Vocational courses (5) and those not attending school (6), and these were categorized into 3; those that had no formal school (0) and those that were attending primary level of education (1) and those that were attending secondary level of education (2). Vocational courses were categorized under secondary level.

The socio-economic status was assessed using a wealth index that assessed ownership of household items as assessed by Filmer et al (29). The participants were asked if they possessed the items or not. A participant was recorded as possessing the item only if the item was functioning. The items included television, electricity, bicycle, water motor car, flush toilet, mobile phone, computer, radio, motorcycle, and refrigerator. Using principal component analysis (PCA), five quantiles were generated with 1 representing the poorest and 5 the richest quintile. These were further categorized into least poor (1) and poorest (0) quintiles. Home description had five options which included very good (1), good (2), bad (3), very bad (4) and I don't know (5). These were categorized into 2; good (1) and bad (2).

Under enabling factors, the questions concerning fear of going to the dentist had 3 options (1–3); I do not fear (1), I fear a little (2) and very fearful (3) and these were re-categorized into 2; “yes (1)” for both little fear and fear and “no (0)” for no fear. The questions concerning fear of HIV spread, avoidance of dental care due to HIV status, avoidance of dental care services due to cost, and failure to receive dental treatment because of other medical conditions had responses with 4 options (1–4) and these included yes, several times (1), yes, a few times (2), no, never (3) and I don’t know (4) and these were categorized into 2; “yes (1) or no (0)”, “yes” for both several times and a few times and “no” for “no, never”. All responses with “I don’t know” were added to the most frequent response.

The question regarding who is responsible for decision making in seeking dental care services had the following responses: parents (1), myself (2), my caregiver (3), my teacher (4) and I do not know (5). These were categorized into 2: parents/teachers/caregivers (0) and myself (1). Under need related factors, rating on general and oral health had five options. These included poor (1), fair (2) good (3), very good (4) and excellent (5). These were further categorized into three; poor (1), fair (2) or good (3). Satisfaction of oral health was assessed via four parameters, and these included very satisfied (1) satisfied (2), dissatisfied (3) and very dissatisfied (4) and these were categorized into two options either satisfied (1) or dissatisfied (0). “Satisfied (1)” category included responses of both very satisfied and satisfied while the “dissatisfied” category included responses of both dissatisfied and very dissatisfied. For the factors under personal oral hygiene and dental practices, the frequency of toothbrushing had 6 responses (1–6). These included the following: never (1), several times a month (2–3 times) (2), once a week (3), several times a week (2–6 times) (4), once a day (5) and 2 or more times a day (6). These were then grouped into two; those who rarely brush (0) and those that brush daily (1).

Items used for tooth brushing /tools for tooth brushing assessed included soap, salt, urine, local herbs, ash, toothpaste, and nothing. These items were renamed in Excel workbook 2003(*xlsx), and each item dichotomized into yes (1) for those who used that item for brushing or no (0) for those who did not use that item.

For those who had been to the dentist, responses concerning last visit to the dentist had 5 options (1–5) and these were the following: less than 6 months ago (1), 6–12 months ago (2), more than 1 year but less than 2 years ago (3), 2–5 years ago (4) and more than 5 years ago (5).These were sub-grouped into those that had visited the dentist less than a year ago (1), between 1–2 years ago (2) or more than 2 years ago (3). Reasons for the last dental visit included mandatory school check-ups or routine check-ups, emergency (tooth injury), emergency (toothache), having tooth (teeth) pulled, filling, root canal or others. were renamed and dichotomized into “yes (1)” for those whose responses were positive for a particular reason or “no (0)” for those with negative responses for a reason.

Duration at the dental facility had 5 options that were categorized into 3: less than 1 hour (1), 1 to 2 hours (2) or 3 and more hours (3). Average travel cost was assessed under 5 options (1–5). The options included the following: less than Ug. Sh. 4,000 (1), between Ug. Sh. 4100–10000 (2), no money spent (3), more than 10000 (4) and I do not know (5). These were categorized into; no money spent (0), less than Ug. Sh. 4,000 (1), between Ug. Sh. 4100–10000 (2) or more than 10000 (3). Attitude of the oral health care providers was also assessed 5 options (1–5) and these included very good (1), good (2), average (3), below average (4) and don't know (5) that were categorized into 3; good (1), average (2) or below average (3). “Very good (1) and good (2)” responses were put under the “good (1)” option.

NB: All “I don’t know” responses were added to the most frequent categories.

### Statistical methods

Data was analyzed using Statistical package Stata/SE 17.0 (30). Categorical variables were summarized as percentages while continuous variables were summarized with mean, medians/range. Cross-tabulations were done using Fischer’s exact test (31). Simple modified Poisson model was used at bi-variate analysis while multivariable modified Poisson regression was used to determine factors associated with ever use of dental care at 95% confidence interval and at 0.05 level of significance. The measure of association was in terms of Incidence Risk Ratio (IRR) from the multivariate modified Poisson model that was interpreted as the prevalence ratio (PR) for this study. At multivariate analysis stage, all variables related to predisposing, enabling, need related factors, oral hygiene, and personal health practices with a more likely association with ever use of dental care (p-value ≤ 0.2) in unadjusted analyses were included in the model.

In addition, the 4 clusters (which represented the 4 different clinics the participants belonged) were adjusted for using clustered robust standard errors incorporated in the Poisson regression model.

## Results

Table 1 depicts the distribution of study variables in total and according to sex. A total of 154 adolescents, aged between 10 and 18 years participated in this study. The median and mean age were 14 years and 14.3 respectively while range was 10 to 18 years. The proportion of males was 57.8% (89/154) versus 42.2% (65/154) of females. Seven percent (11/154) of the participants reported having no formal education. More females were attending primary level of education compared to their male counterparts 66.2% (43/65) versus 46.1% (41/89). Overall, 58.4% (90/154) of the participants were in the low quintile category with more males coming from the poorest families compared to females, 64.0% versus 50.8%. In general, most participants (88/154) described their homes as being in good condition. The prevalence of ever-use of dental care among the study participants was 12.3% (19/154).

Table 2 depicts bivariate analysis of the predisposing factors of ever use of dental care services in terms of socio-demographic characteristics. As shown, at cross-tabulation, ever use of dental care services did not associate significantly with any of the predisposing factors despite the remarkable differences between the groups.

According to Table 3, ever use of dental care services was statistically significantly associated with avoidance of dental care services due to the HIV status, the fear of HIV spread, failure to get dental treatment because it is not part of the medical treatment and illness from other medical conditions. Twenty-one percent versus 78.9% (p = 0.01) that confirmed and disconfirmed avoidance of dental care services due to the HIV status respectively reported ever-use of dental care services. Among participants that reported avoidance of dental care services due to fear of spread of HIV, 10.5% versus 89.5% (p = 0.04) that respectively confirmed and disconfirmed the above reported ever-use of dental services. Seventy-nine percent versus 21% (p = 0.00) participants who respectively, disconfirmed and confirmed failure to get dental treatment because it is not part of the medical treatment. In addition, 78.9% versus 21.1% (p = 0.00) of participants who respectively, disconfirmed and confirmed illness from other conditions reported ever use of dental care. Other enabling factors in terms of being afraid of going to the dentist, and the knowledge of a dental facility next to their residence and HIV centre, avoidance of dental care services due to cost and responsible person in decision making were not statistically significantly associated with ever use of dental care services.

As depicted in Table 4, ever use of dental care was statistically significantly associated with the following need related factors, perception of overall health in general, perception of health of teeth and mouth, satisfaction with health of mouth and teeth. Totals of 36.8%, 26.3% and 36.8% of participants having bad, fair, and good perceptions of health of teeth and mouth reported ever use of dental care services respectively (p = 0.00). Totals of 21.1% versus 78.9% (p = 0.02) being respectively satisfied and dissatisfied with health of mouth and teeth confirmed ever use of dental care services. Under perception of general health, totals of 15.8%, 31.6% and 52.6% of participants reported having respectively bad, fair, and good perceptions of health of teeth and mouth reported ever use of dental care services (p = 0.04). As seen with the unadjusted analysis, need for dental treatment, dental and mouth pain, bleeding gums, and bad oral odour were not statistically significantly associated with ever-use of dental care services.

### Personal oral hygiene and dental practices

As shown in Table 5, when participants were assessed for personal oral hygiene and dental practices, unadjusted analysis showed that, none of the personal oral hygiene and dental practice was statistically significantly associated with ever-use of dental care services.

### Multivariate analysis

Table 6 summarizes the regression analysis of ever use of dental care services using modified Poisson regression models. Participants aged 14–18 years were 3.35 times more likely to report ever-use of dental care services compared to those who belonged to the 10–13 age category (PR 3.35, 95% CI 1.48–7.59). Compared to participants that were not afraid of going to the dentist, those who were afraid were 2.98 times more likely (PR 2.98, 95% CI 1.41–6.30) to report ever use of dental care services. The participants that reported avoidance of dental care services due to fear of spread of HIV were 0.94 times less likely to report ever-use of dental care services (PR 0.06 95% CI 0.01–0.44). On the other hand, those that reported yes to lack of co-ordination of dental care appointments with medical appointments were 4.50 times more likely to report ever use of dental care services compared to their counterparts who said no (PR 4.50 95% CI 1.14–17.80). In comparison to participants who were not satisfied with their dental condition, those that were satisfied with their dental condition were 0.79 times less likely to report ever-use of dental care services (PR 0.21 95% CI 0.05–0.94). Participants who reported bad oral odour were 2.8 times more likely to report ever-use of dental care services compared to their counterparts who did not report bad oral odour (PR 2.80 95% CI 1.19–6.60). In reference to participants who did not use soap for toothbrushing, those who used soap were 2.51 times more likely to report ever-use of dental care services (PR 2.51, 95% CI 1.47–4.28). In addition, gender, perception about health of teeth and mouth, perception about overall general health, avoidance of dental care services due to HIV status, avoidance of dental care due to cost, and illness from other medical conditions were not statistically associated with ever-use of dental care services. The multivariate model was tested for goodness-of fit using a pseudo -R squared value.

#### Frequency distribution of oral health and related characteristics of those who had ever visited the dentist

As illustrated in Table 7, the main reason for the last dental visit was tooth extraction (“having teeth pulled”) with 15 out of 19 participants. Emergency toothache was reported by 12 out of 19 participants. Other reasons mentioned included Root Canal treatment (1/19) and filling (3/19). A majority, 12 out 19 participants sought dental care services and only 7 out of 19 participants sought dental care from private facilities. Majority of the participants used buses/taxis as transport to the dental facilities (8/19). Most participants reported spending more than three hours at the dental facility when they sought dental treatment (13/19). When asked about the average travel cost to the dental facility, most participants reported it was between 4,100 and 10,000 Uganda shillings (9/19). Fifteen participants out of 19 reported that they felt that the dental team listened well and gave time to listen to their oral problems. One of 19 of the participants reported withholding their serostatus from the dental health care provider. About 2/19 of the participants were dissatisfied with the quality of dental care rendered to them due to their serostatus. Three out of 19 of the participants felt that the quality of dental care services rendered to them was different from their seronegative counterparts. 12 out of 19 participants had a good perception on the dentist that offered dental treatment while 3/19 had a poor perception on the dentist that offered dental treatment.

## Discussion

The study found that Ugandan adolescents aged 10–18 use dental care services slightly above 10%, influenced by factors like predisposing, enabling, need-related, and personal oral hygiene practices as defined by Andersen’s behavioral model (28). The study found that age, fear of dental visits, avoidance of dental care due to fear of HIV spread, and failure to get dental treatment due to not being part of medical appointments were significant enabling factors for ever-use of dental care services. However, satisfaction with dental condition and bad oral odor were also associated with ever-use. The Andersen's behavioral model explained 26% of the variance in dental care use, with enabling factors being more significant. Personal oral hygiene and dental practices were of the least importance in relation to ever-use of dental care services.

The study strengths included; it explored the use of dental care services among vulnerable adolescents living with HIV on ART in Uganda, a population where such information is scarce. It used interviews to gather data, avoiding confusion and allowing for easier recruitment and data collection, as part of a larger parent study (32). The study utilized mobile phones to address sensitive HIV and dental care questions without victimization, promoting privacy and potentially increasing response rates(33). The Open data kit minimized human error during data collection through authentication, eliminating item non-response (34). The study also used novel statistical methods to minimize model misspecification bias (35). The study found that ever-use of dental care services is a less seldom outcome, and the commonly used ordinary logistic regression could have overestimated associations. Therefore, Poisson regression was preferred over ordinary logistic regression and modified to use robust standard errors for binary outcomes (35, 36). The study used Andersen's behavioral model as a theoretical framework, ensuring the validity of the sub-study variables. The 26% variance indicated a good fit for the data, but it also explains other variables as important determinants of dental care use among HIV adolescents.

Nevertheless the study had some limitation including; the study findings could have been skewed by potential confounding factors like the nature of study design as it did not establish causal relationships but rather risk indicators (37). The use of Andersen's behavioral model had limitations, including lack of standardized variables making it less suitable for comparison with other studies (38). The study's findings may have been influenced by recall bias (39), social desirability bias, selection bias, and insufficient sample size. Participants were asked about ever-use of dental care services, which could have led to misunderstandings about childhood experiences. Previous exposure to free dental screening and emergency treatment by study participants may have overestimated the prevalence of ever-use. The study's sampling process, insufficient sample size, and unit non-response may have compromised the accuracy of the findings. Additionally, the study's selection bias could have been influenced by the inclusion of a limited number of participants, potentially compromising the precision of the findings (40). The study's generalizability to a larger population of adolescents with HIV and ART in Kampala or Uganda may be limited due to insufficient sample size and unit non-response. However, the selection of HIV centres was based on ART coverage (41).

This study found that 12.3% of HIV-positive adolescents reported ever-use of dental care services, slightly lower than a 19% rate in a study in China (42). The differences could be attributed to differences in economic situations, dental accessibility, and health financing systems in the different regions (43, 44). Other studies in Nigeria, Uganda, and Tanzania reported rates of dental care utilization of 8%, 9%, and 18.5%, respectively (14, 45, 46). Likened to this study, a US study that found older age groups more likely to be retained in oral health care (47). Inconsistent with this study, previous studies have showed that fear of dentists is a barrier to dental care services for adults living with HIV (45, 48-50). This may be due to age and cultural differences. Similar to this study, a Sudan study reported that 75% of dental patients fear HIV transmission at dental facilities (51). A study in Florida found that adults with HIV experienced treatment fatigue due to overwhelming appointments with doctors and dentists (49). Another study in Northern California found 21% of women living with HIV failed to book dental appointments and poor oral health perception had a negative influence on use of dental care services (50). Studies have shown that dental condition satisfaction positively influences dental care service use in the general adult population of unknown HIV status, not necessarily the HIV adolescent population (52). Studies in South Africa and Kenya show that most children living with HIV have extractions for dental caries management, with emergency toothache being the primary reason for visiting the dentist (53, 54). In a Florida study, most participants noted spending a lot of time at the dental facility, with long waiting times for appointments with discrimination concerns due to the HIV status (49).

Some participants felt dental care services were different from non-HIV friends and families, and dentists treated them poorly with one withholding her HIV status. This study is similar to a Sudanese study where 50.4% of participants had a negative attitude towards people with HIV, primarily concerned about HIV transmission (51).

This study reveals that HIV positive adolescents in Kampala face limited access to dental care services which may be due to limited awareness and limited availability of dentists (18). Many patients may be unaware of the dental services due to a lack of an HIV dental integrated program in Uganda. Additionally, dental clinics are often privately owned, making them expensive for adolescents living with HIV (19). The perception of premature death or a short life span also contributes to low levels of dental care use (49). Concerning age, the older the individual, the longer they are exposed to the adverse effects of ART, potentially leading to high dental caries rates (17). Most participants only visit the dentist as an emergency, and fear of dental visits may also contribute to poorer oral health hence greater need for dental services (55, 56). The fear of HIV spread in dental facilities may be attributed to a lack of trust in infection control systems. Studies show that not washing hands, using protective eyewear, and sterilizing instruments can negatively affect dental care use (57). Participants may not be knowledgeable about infection control protocols. The study found a lack of coordination between medical and dental appointments. The findings suggest that dental care services are primarily utilized for urgent dental needs, despite the lack of coordination between appointments among participants. This could be coupled with the frequent medical care required for HIV patients, making it difficult to balance oral health needs. The time spent at the dental facility could negatively impact school attendance and income for caregivers and parents. The study found that participants satisfied with their oral condition were less likely to use dental care services, possibly due to lack of pain or toothache. Conversely, bad oral odor negatively affects HIV patients' quality of life, possibly due to ART-induced xerostomia and oral lesions (13, 58). The study findings may suggest that soap lacks fluoride hence can’t prevent dental caries, leading to increased dental service utilization in patients due to the frequency of dental caries in participants that use soap. However, participants may be motivated to brush their teeth, and due to toothpaste costs, they adopt soap as an alternative. The study reveals that many individuals in Uganda are unaware of treatment options and limited access to preventive treatments, leading to a lack of regular dental care services. This is due to financial status and perception of the relevance of dental treatment. Dental and mouth pain often presents as emergencies, leading to a focus on treatment rather than prevention (59). This low priority for oral health negatively affects quality of life, leading to delayed dental visits. The study also found that the cost of dental care services significantly influences the type of facility participants visit. Most dental patients sought care from public facilities and used public transport, which is a cheap alternative for many Ugandans. This could be a barrier to seeking dental services in urban settings like Kampala, as low social economic status individuals in the are more likely to be infected with HIV (60). Long-waits at the dental visit may be due to the large patient-provider ratio. The study reveals that despite HIV education, stigma and discrimination persist among HIV-positive individuals, leading to a negative attitude from dental care providers. This fear of rejection and confidentiality may deter them from seeking necessary dental services. The findings provide a basis for further research on HIV-related dental experiences in Uganda.

The study suggests the need for public health campaigns to raise patient dental awareness, highlight availability and usage of oral health programs, and reduce fear about dental care (48, 61). It also emphasizes the importance of good oral health and care for persons living with HIV. Synchrony between medical and dental appointments is crucial for comprehensive management. More studies are needed to determine the true association between demographics and dental care use. Investing in dental practitioner training and conducting research on HIV stigma and its association with dental care services is crucial for addressing dental needs of adolescents living with HIV (62).

## Conclusion

The study found low frequency of dental care use among HIV infected adolescents in Kampala, Uganda, with age being a predisposing factor. Enabling factors included fear of HIV spread, dental appointment failure, and satisfaction with dental condition and bad oral odor while under personal oral hygiene and dental practices, use of soap for toothbrushing was an important association of use of dental care.

## Figures and Tables

**Table 1:** Distribution of study variables in total and by sex among10-18-year-old adolescents (n = 154)

Variables	Total (n = 154)	Male (n = 89)	Female (n = 65)
**Continuous variables Median (IQR)**			
Age (years)	14(10–18)	14(10–18)	14(10–18)
**Categorical Variables frequency (%)**			
**Age categories**			
10–13	59(38.3)	34(38.2)	25(38.5)
14–18	95(61.7)	55(61.8)	40(61.5)
**Education level**			
No formal school	11(7.1)	8(9.0)	3(4.6)
Primary level	84 (54.6)	41(46.1)	43(66.2)
Secondary level	59 (38.3)	40 (44.9)	19 (29.2)
**Socioeconomic status**			
Low quintile	90(58.4)	57(64.0)	33(50.8)
High quintile	64(41.6)	32(36.0)	32(49.2)
**Home description**			
Good	88(57.1)	47(52.8)	41(63.1)
Bad	66(42.9)	42(47.2)	24(36.9)
**Ever-use of dental care**			
No	135 (87.7)	78 (87.6)	57 (87.7)
Yes	19 (12.3)	11 (12.4)	8 (12.3)

**Table 2:** Percentages (n) of adolescents, 10–18 years confirming ever use of dental care services according to predisposing factors. Fisher’s exact test, (n = 154)

Variable	Ever visited the dentist		
	Yes n (%)	No n (%)	P value
**Gender**	11(57.9)	78 (57.8)	1.00
Male	8(42.1)	57 (42.2)	
Female			
**Age**	5 (26.3)	54 (40.0)	0.32
10–13			
14–18	14(73.7)	81 (60.0)	
**Education level**			
No formal school	1 (5.3)	10 (7.4)	0.14
Primary level	7 (36.8)	77 (57.0)	
Secondary level	11 (57.9)	48 (35.6)	
**Socio-economic status**			
Low quintile	11 (57.9)	79 (58.5)	1.00
High quintile	8 (42.1)	56 (41.5)	
**Home description**			
Good	12(63.2)	76 (56.3)	0.63
Bad	7(36.8)	59 (43.7)	

**Table 3:** Percentages (n) of participants confirming ever use of dental care services according to enabling factors, Fisher’s exact test, (n = 154)

Variable	Ever visited the dentist		
	Yes n (%)	No n (%)	P- value
**Afraid going to dentist**			
No	15(78.9)	121 (89.6)	
Yes	4(21.1)	14 (10.4)	0.24
**Knowledge of Dental facility next to residence**			
No	12 (63.2)	93 (68.9)	
Yes	7(36.8)	42 (31.1)	0.61
**Knowledge of Dental facility next to HIV Centre**			
No	5(26.3)	39 (28.9)	
Yes	14(73.7)	96(71.1)	1.00
**Have you ever avoided dental care due to your HIV status?**			
No	15(78.9)	131 (97.0)	0.01
Yes	4(21.1)	4(3.0)	
**Have you avoided dental care due to fear of spread of HIV?**			
No	17 (89.5)	134 (99.3)	0.04
Yes	2 (10.5)	1 (0.7)	
**Failure to get dental treatment because it is not part of the medical appointment**			
No	15(78.9)	135 (100.0)	
Yes	4(21.1)	0 (0.0)	0.00
**Illness from other conditions**			
No	15(78.9)	132(97.8)	
Yes	4(21.1)	3 (2.2)	0.01
**Have you ever avoided dental care due to cost?**			
No	14(73.7)	117 (86.7)	
Yes	5(26.3)	18 (13.3)	0.17
**Responsible person in decision making**			
Parents/caregiver/teacher	18 (94.7)	116 (85.9)	
Myself	1 (5.3)	19 (14.1)	0.47

**Table 4:** Percentages (n) of adolescents 10–18 years confirming ever use of dental care services according to need related factors. Fisher’s exact test (n = 154)

Variable	Ever visited the dentist		P value
	Yes n (%)	No n (%)	
**Perception about overall general health**			
Poor	3 (15.8)	5 (3.7)	
Fair	6 (31.6)	30 (22.2)	
Good	10 (52.6)	100 (74.1)	0.04*
**Perception about health of teeth and mouth**			
Bad	7(36.8)	9 (6.7)	
Fair	5(26.3)	55 (40.7)	
Good	7(36.8)	71 (52.6)	0.00**/0.002
**Satisfaction teeth**			
Dissatisfied	15(78.9)	66 (48.9)	
Satisfied	4(21.1)	69 (51.1)	0.02*
**Need for dental treatment**			
No	4(21.1)	41 (30.4)	
Yes	15(78.9)	94 (69.6)	0.59
**Dental and mouth pain**			
No	9(47.4)	79 (58.5)	0.46
Yes	10(52.6)	56 (41.5)	
**Bleeding gums**			
No	13(68.4)	104 (77.0)	0.40
Yes	6(31.6)	31 (23.0)	
**Bad oral odour**			
No	13(68.4))	112 (83.0)	0.21
Yes	6(31.6)	23 (17.0)	

NB: At bivariate analysis; **p < 0.001, *p < 0.05

**Table 5:** Percentages (n) of adolescents, 10–18 years, confirming ever-use of dental care services according to personal oral hygiene and dental practices, Fisher’s exact test, (n = 154)

Variable	Ever visited the dentist		P -value
	Yes n (%)	No n (%)	
**Tooth brushing frequency**			
Rarely brush	7 (36.8)	56 (41.5)	
Daily	12(63.2)	79 (58.5)	0.81
**Oral hygiene materials**			
**Use of soap**			
No	15(78.9)	125 (92.6)	
Yes	4 (21.1)	10 (7.4)	0.07
**Use of salt**			
No	17(89.5)	107 (79.3)	
Yes	2 (10.5)	28 (20.7)	0.37
**Use of local herbs**			
No	19 (100.0)	132 (97.8)	
Yes	0 (0.0)	3 (2.2)	1.00
**Use of Ash**			
No	16(84.2)	104 (77.0)	
Yes	3 (15.8)	31 (23.0)	0.57
**Use of Toothpaste**			
No	1(5.3)	3 (2.2)	
Yes	18(94.7)	132 (97.8)	0.41

**Table 6:** Predisposing, enabling, need related factors, personal oral hygiene and dental practices associated with ever use of dental care services among HIV positive adolescents 10–18 years. Modified Poisson regression analysis (n = 154)

Variable	PR	95%CI	P-value
***Predisposing factors***			
*Gender*	1	1	0.28
Male	1.43	0.74–2.78	
Female			
*Age*	1	1	0.04
10–13	3.35	1.48–7.59	
14–18			
**Enabling factors**			
*Afraid of going to the dentist*	1	1	0.04
No	2.98	1.41–6.30	
Yes			
*Have you ever avoided dental care due to your HIV status?*	1	1	0.08
No	1.87	0.92–3.80	
Yes			
*Have you avoided dental care due to fear of spread of HIV?*	1	1	0.01
No	0.06	0.01–0.44	
Yes			
*Have you ever avoided dental care due to cost?*	1	1	0.27
No	0.61	0.25–1.45	
Yes			
*Failure to get dental treatment because it is not part of the medical appointment*	1	1	0.03
No	4.50	1.14–17.80	
Yes			
*Illness from other conditions*	1	1	0.10
No	8.21	0.70–96.90	
Yes			
**Need related factors**			
*Perception about health of teeth and mouth*	1	1	0.14
Bad	0.18	0.02–1.77	0.79
Fair	1.08	0.60–1.95	
Good			
*Perception about overall general health*	1	1	0.59
Bad	0.82	0.39–1.72	0.06
Fair	0.55	0.30–1.01	
Good			
*Satisfaction with teeth condition*	1	1	0.04
No	0.21	0.05–0.94	
Yes			
*PR- Prevalence Ratio CI- Confidence Interval*			
*Bad oral odour*	1	1	0.02
No	2.80	1.19–6.60	
Yes			
**Personal oral hygiene and dental practices**			
**Oral hygiene materials**			
*Use of soap*	1	1	0.00
No	2.51	1.47–4.28	
Yes			
*PR- Prevalence Ratio CI- Confidence Interval*			

**Table 7:** Percentage (n) distribution of oral health and related characteristics among those who had ever visited a dentist (n = 19)

Variable	Frequency n (%)
***Reason for the last dental visit***	
***Mandatory school check up***	
No	18 (94.7)
Yes	1 (5.3)
**Emergency toothache**	
No	7 (36.8)
Yes	12 (63.2)
**Having teeth pulled (tooth extraction)**	
No	4 (21.1)
Yes	15 (78.9)
**Filling**	
No	16 (84.2)
Yes	3 (15.8)
**Root canal**	
No	18 (94.7)
Yes	1 (5.3)
**Others**	
No	18 (94.7)
Yes	1 (5.3)
**Last dental visit**	
Less than 1 year	13 (68.4)
More than 1 year but less than 2 years	4 (21.1)
More than 2 years	2 (10.5)
**Site of dental services**	
Private	7 (36.8)
Public	12 (63.2)
**Means of transport to the dental facility**	
Walking	5 (26.3)
Bus/taxi	8 (42.1)
Bicycle	2 (10.5)
Motorcycle	4 (21.1)
**Time at the dental facility**	
Less than 1 hour	2 (10.5)
Between 1 hour to 2 hours	4 (21.1)
More than 3 hours	13 (68.4)
**Travel cost to the dental facility**	
No money spent	2 (10.5)
Less than Ug. Sh. 4,000	4 (21.1)
Between Ug. Sh. 4100–10000	9 (47.4)
More than 10000	4 (21.0)
**Did you feel the dental team listened well and gave you time to explain your problem**?	
No	4 (21.1)
Yes	15 (78.9)
**Has the dentist ever asked your HIV serostatus before offering dental treatment?**	
No	14 (73.7)
Yes	5 (26.3)
**Have you ever hidden/withheld your HIV status from the dentist before receiving dental treatment?**	
No	18 (94.7)
Yes	1 (5.3)
**Have you ever felt not satisfied with the dental care you received because of your HIV status?**	
No	17 (89.5)
Yes	2 (10.5)
**Have you ever felt the dental care rendered to you was different from your friends and family whom you think do not have HIV?**	16 (84.2)
No	3 (15.8)
Yes	
**In general, how would you describe the way your dentist treated you?**	3 (15.8)
Poor	4 (21.0)
Average	12 (63.2)
Good	

## List Of Abbreviations

AIDS: Acquired Immunodeficiency Syndrome
ART: Antiretroviral treatment
DMFT: dmft Decayed, missing, filled teeth
HIV: Human Immunodeficiency Virus
KCCA: Kampala Capital City Authority
MJAP: Makerere University Joint AIDS Program
NUG: Necrotizing Ulcerative Gingivitis
NUP: Necrotizing Ulcerative Periodontitis
PCA: Principal Component Analysis
PLHIV: People Living with HIV
UPHIA: The Uganda Population-Based HIV Impact Survey
UNAIDS: Joint United Nations Program on HIV and AIDS
IRR: Incidence Rate Ratio
PR: Prevalence Ratio
CI: Confidence Interval
MakSHSREC: Makerere University Institutional Review Board, School of Health Sciences Research and Ethics Committee
UNCST: Uganda National Council of Science and Technology
REK: Norwegian Regional Ethics Committee
